# Editorial: Repair and regenerative mechanisms by stem/progenitor cells and secretome: The golden twenties of translational medicine

**DOI:** 10.3389/fmed.2022.901003

**Published:** 2022-08-08

**Authors:** Roberto Gramignoli, Fabio Sallustio, Darius Widera

**Affiliations:** ^1^Laboratory Medicine, Karolinska Institutet, Solna, Sweden; ^2^Karolinska University Hospital, Huddinge, Sweden; ^3^Department of Interdisciplinary Medicine, University of Bari Aldo Moro, Bari, Italy; ^4^MIRROR-Medical Institute for Regeneration, Repairing and Organ Replacement, Interdepartmental Center, University of Bari Aldo Moro, Bari, Italy; ^5^University of Reading, Reading, United Kingdom

**Keywords:** cell-based medicinal products, secretome, translational applications, regenerative medicine, secreted metabolites

The years enclosed between 1920 and 1929 have frequently been referred to as the “Golden Twenties” in Europe (because of the economic boom following WWI). This third decade of the new millennium may optimistically be a golden time in translational medicine, as researchers are able to explore potential in healthcare as well as out in biotech enterprises. Regenerative medicine is a fast-growing branch of translational research, continuously providing new concrete examples of biotherapeutics and bio-engineered strategies. Within this second edition of the Research Topic in “*Tissue Repair and Regenerative Mechanisms by Stem/Progenitor Cells and their Secretome”* we have collected examples of ongoing translational biotherapies and offered expert glimpses into future medications. Experts in the field have illustrated examples of individualized regenerative approaches, where different cellular or molecular mediators can offer correction rather than amelioration.

Historically, modern medicine has been limited to treatment of symptoms of congenital or chronic disorders, with only a few exceptions such as hormone replacement therapy and antimicrobials. The alleviation of the symptoms has immediate effects on the patient, resulting in a positive outcome and enhanced welfare, but it does not eradicate the cause or ameliorate prognosis. Whenever possible, healthcare should also include a component of disease correction—an approach aiming to resolve rather than attenuate disease progression. In this new era, we are refining and optimizing a growing armamentarium of therapeutic options that may significantly impact patients affected by both congenital and chronic disease.

The translational approaches described in this SI have been offering both replacement of missing functions as well as removal of pathophysiological blocks that prevent spontaneous regeneration. The first study submitted and accepted for the current Research Topic on “*Tissue Repair and Regenerative Mechanisms by Stem/Progenitor Cells and their Secretome”* details the effects offered by human hemangioblast-derived mesenchymal stromal cells (MSC) in support or as trophic mediator to pancreatic islets. The co-transplantation of murine islets shielded with third-party hemangioblast-MSC restored glycemic control in diabetic J:Nu nude animals. Such *in vivo* analyses have been coupled with *in vitro* measurements describing reduction of cytokine stress (Bertera et al.).

Similar immunological protection and trophic support was described in skin allograft. In this study, the third-party stem cells are epithelial stem cells isolated from human placenta. Such cells were administered intravenously or subcutaneously in support of skin allograft (mouse-into-mouse) or xenograft (rat-into-mouse). Human stem cells have been pre-activated with pro-inflammatory cytokines (IL-1β and INFγ), and reported to be efficient in preventing allo- or xeno-rejection (equally efficient to classical pharmacological transdermal injection) (Kolanko et al.).

The same Polish group contributed a second research article, where Laminin isoform 332 was tested as substrate for amnion-derived stem cells. Such purified extracellular protein preserved viability and identity of primary cells maintained for a short time in culture. The authors measured markers for pluripotency and early differentiation, as well as immunomodulatory and adhesion capacities (Skowron-Kandzia et al.).

Interestingly, amnion membrane is the tissue of origin for cells included in another study included in this Research Topic: Lo Nigro et al. evaluated and reported therapeutic effects of amnion-derived MSC in end-stage liver disease settings. The authors examined intact cells but also secreted mediators, reporting how these soluble components reduce cell death but also enhance liver regeneration in response to a lethal hepatic insult (Lo Nigro et al.).

Another manuscript exploiting the potential of perinatal MSC has been included in the current SI, where cells isolated from umbilical cord, rather than amnion membrane, have been used to treat a patient affected by COVID-19. Zhang Q. et al. reported anti-inflammatory and immune-modulatory effects offered by human MSC, reversing acute respiratory syndrome. Such clinical outcome has been proven by improvement in inflammatory interleukins IL-4, IL-6, and IL-10 (Zhang Q. et al.).

Regeneration and repair can proceed *via* many processes in different tissues. Both direct cell differentiation and paracrine soluble mediators have been shown to serve in reducing damaged areas and facilitating survival of resident cells. And secreted vesicles are the main topic of a review article detailing the therapeutic effects of amnion membrane-derived secretome. Fathi and Miki compiled an overview of the current scientific literature on the different components and constituents of amnion secretome, as well as their effects in different clinical conditions (Fathi and Miki).

A systematic revision has also been compiled on the treatment of bone fracture using platelet-rich plasma, with particular attention to criticism and conflicting results. Both preclinical and clinical reports have been reviewed reporting the application of platelet-enriched solutions as a promising therapy for bone fracture (Zhang Y. et al.).

The third review manuscript also focuses on the musculoskeletric system, with an analogous supportive approach. Li et al. described platelet-rich plasma application in tendon injuries (Li et al.).

Natural evolution has applied strong survival pressure and selection of cellular mechanisms to limit wound severity. Daily we experience physiological pressures generating “healing” processes aimed to rapidly close wounds and limit damage, with little or no regard for long-term function of the damaged tissue. The replacement of functional tissue with scars as well as chronic damage result in loss of tissue or whole organ function. Such loss frequently impairs overall survival and always impacts quality of life. Drastic treatments, such as whole organ transplantation, are frequently the sole therapy for severely compromised systems. But organ transplantation is not a cure, it is only a treatment leading to a milder disease.

Since stem cell therapy has the capacity to influence all the processes described above, scientific interest is convening toward new stem cell strategies and their translation to the clinic.

## Author contributions

RG compiled first and final version of the manuscript. FS and DW revised the manuscript. All authors contributed to the article and approved the submitted version.

## Conflict of interest

The authors declare that the research was conducted in the absence of any commercial or financial relationships that could be construed as a potential conflict of interest.

## Publisher's note

All claims expressed in this article are solely those of the authors and do not necessarily represent those of their affiliated organizations, or those of the publisher, the editors and the reviewers. Any product that may be evaluated in this article, or claim that may be made by its manufacturer, is not guaranteed or endorsed by the publisher.

